# Supplementary figures and images for: Aging is associated with increased brain iron through cortex-derived hepcidin expression
Source: eLife. 2022 Jan 11;11:e73456. doi: 10.7554/eLife.73456 (PMC8752087; doi:10.7554/eLife.73456)

## Slide 1
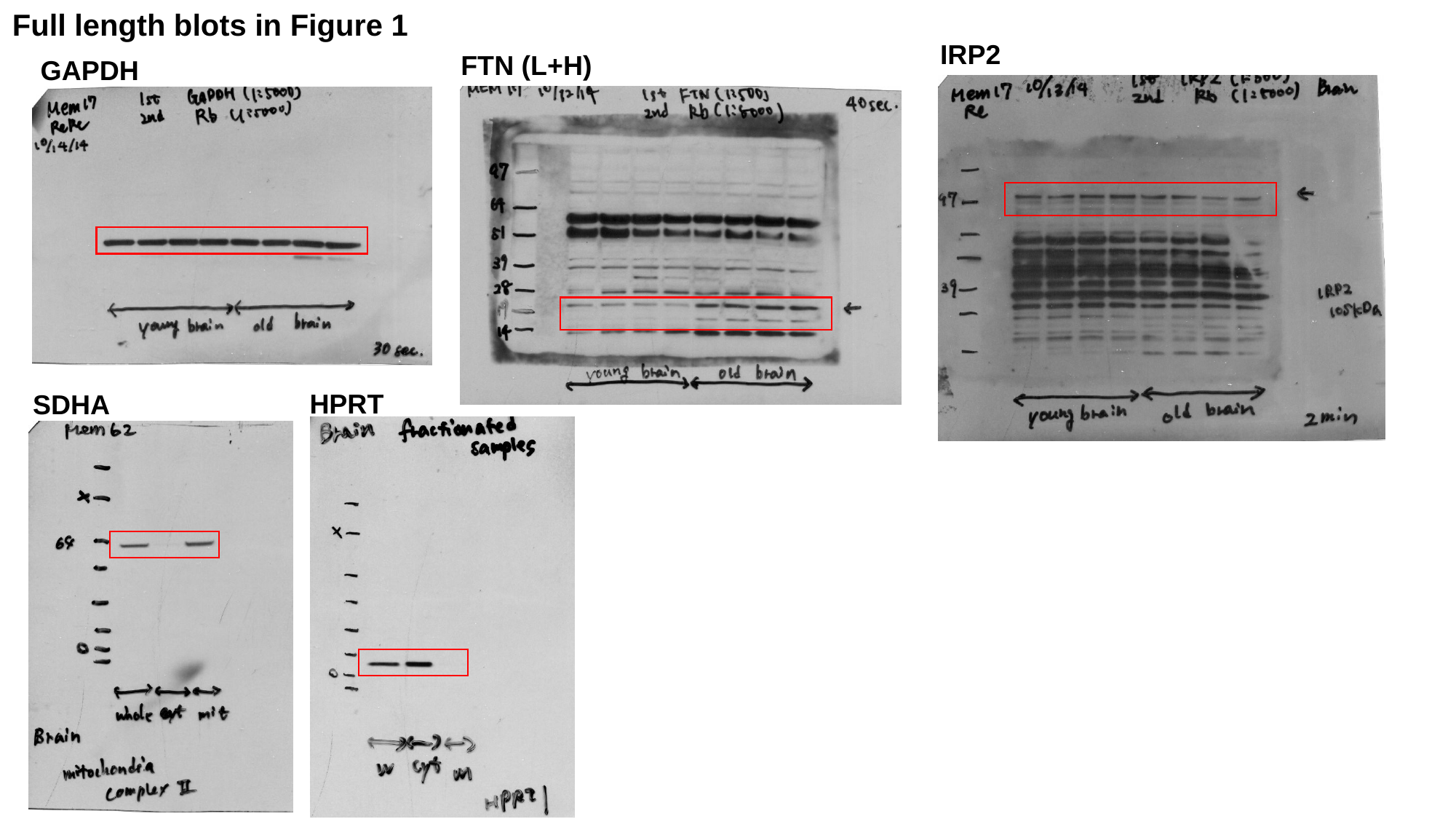

Full length blots in Figure 1
IRP2
FTN (L+H)
GAPDH
HPRT
SDHA

Supplement: Figure 1—source data 1. [file elife-73456-fig1-data1.pptx]
