# Supplementary material for: Aging is associated with increased brain iron through cortex-derived hepcidin expression
Source: eLife. 2022 Jan 11;11:e73456. doi: 10.7554/eLife.73456 (PMC8752087; doi:10.7554/eLife.73456)
Supplement: Figure 2—source data 1. [file elife-73456-fig2-data1.pptx]

## Slide 1
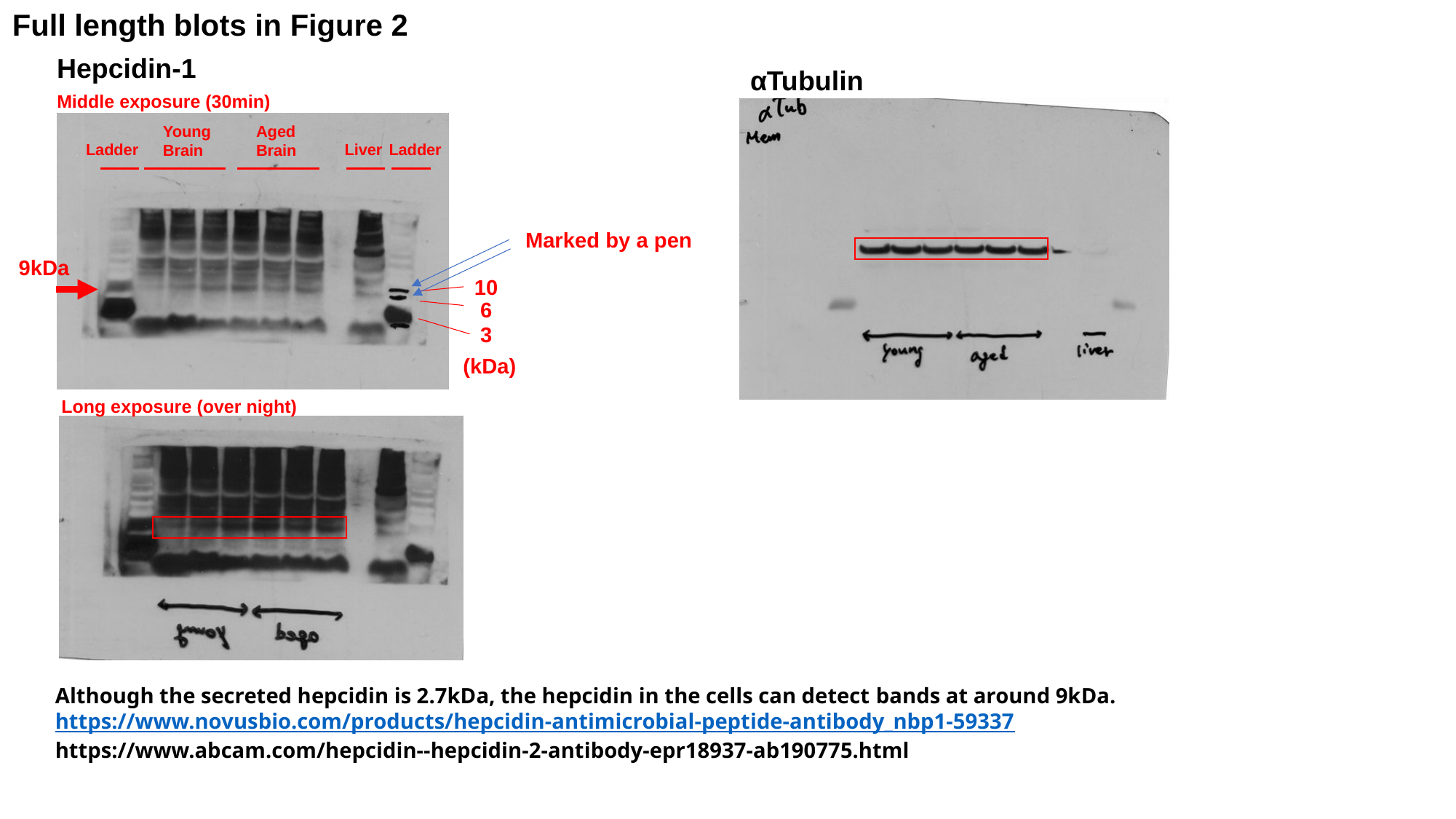

Full length blots in Figure 2
Hepcidin-1
αTubulin
Middle exposure (30min)
Young
Brain
Aged
Brain
Ladder
Liver
Ladder
Marked by a pen
9kDa
10
6
3
(kDa)
Long exposure (over night)
Although the secreted hepcidin is 2.7kDa, the hepcidin in the cells can detect bands at around 9kDa.
https://www.novusbio.com/products/hepcidin-antimicrobial-peptide-antibody_nbp1-59337
https://www.abcam.com/hepcidin--hepcidin-2-antibody-epr18937-ab190775.html
