# Supplementary material for: Aging is associated with increased brain iron through cortex-derived hepcidin expression
Source: eLife. 2022 Jan 11;11:e73456. doi: 10.7554/eLife.73456 (PMC8752087; doi:10.7554/eLife.73456)
Supplement: Figure 3—source data 1. [file elife-73456-fig3-data1.pptx]

## Slide 1
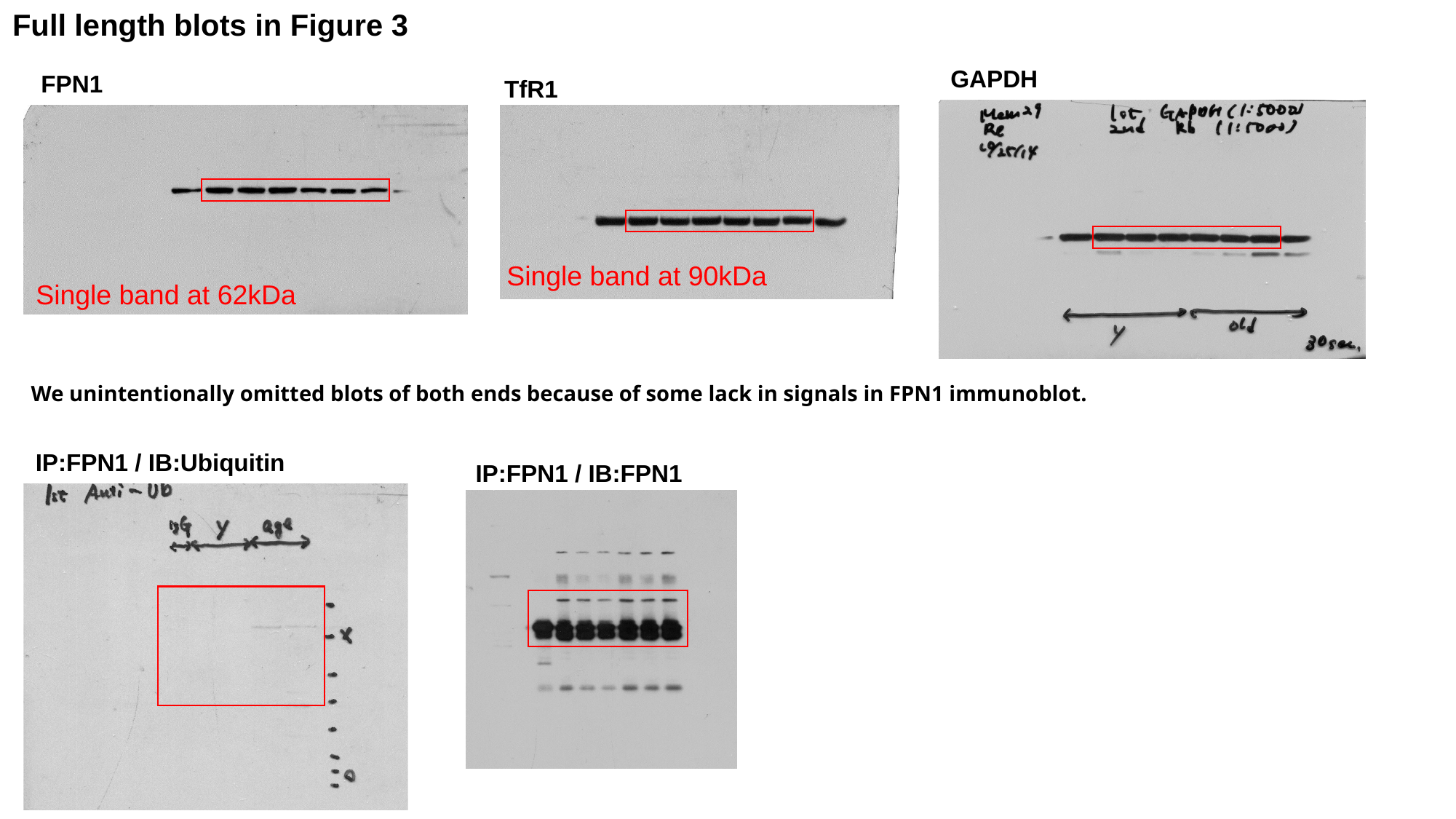

Full length blots in Figure 3
GAPDH
FPN1
TfR1
Single band at 90kDa
Single band at 62kDa
We unintentionally omitted blots of both ends because of some lack in signals in FPN1 immunoblot.
IP:FPN1 / IB:Ubiquitin
IP:FPN1 / IB:FPN1
